# Investigating the Structure and Properties of Epoxy Nanocomposites Containing Nanodiamonds Modified with Aminoacetic Acid

**DOI:** 10.3390/polym16040449

**Published:** 2024-02-06

**Authors:** Anton Mostovoy, Amirbek Bekeshev, Andrey Shcherbakov, Lyazzat Tastanova, Marzhan Akhmetova, Ainagul Apendina, Marina Lopukhova

**Affiliations:** 1 Laboratory of Modern Methods of Research of Functional Materials and Systems, Yuri Gagarin State Technical University of Saratov, Polytechnichskaya Str., 77, 410054 Saratov, Russia; 2 Laboratory of Polymer Composites, K. Zhubanov Aktobe Regional State University, Aliya Moldagulova Avenue 34, Aktobe 030000, Kazakhstan; amirbek2401@gmail.com; 3 Laboratory of Support and Maintenance of the Educational Process, Yuri Gagarin State Technical University of Saratov, Polytechnichskaya Str., 77, 410054 Saratov, Russia; gassmed7@gmail.com; 4 Department “Chemistry and Chemical Technology”, K. Zhubanov Aktobe Regional State University, Aliya Moldagulova Avenue 34, Aktobe 030000, Kazakhstan; lyazzatt@mail.ru (L.T.); k.ajnagul@mail.ru (A.A.); 5 Department “Physics”, K. Zhubanov Aktobe Regional State University, Aliya Moldagulova Avenue 34, Aktobe 030000, Kazakhstan; majiko.a@gmail.com; 6 Department of Economics and Humanitarian Sciences, Yuri Gagarin State Technical University of Saratov, Polytechnichskaya Str., 77, 410054 Saratov, Russia; mlopuhova@yandex.ru

**Keywords:** epoxy oligomer, filler, nanodiamonds, functionalization, modification, aminoacetic acid, physicochemical and mechanical properties

## Abstract

This paper presents a study on the prospects of functionalizing nanodiamonds (NDs) with aminoacetic acid to obtain high-strength composites based on an epoxy matrix. The impact of the functionalization of the ND surface with aminoacetic acid in various concentrations on the properties of the epoxy composite was assessed. The success of grafting amine onto the ND surface was confirmed by X-ray phase analysis and IR spectroscopy. The results show a significant decrease in the average size of ND particles, from 400 nm for the pristine ones to 35 nm, and the contact angle, from 27° to 22°, with an increase in the specific surface area after treatment with a 5% solution of aminoacetic acid. Reducing the average size of NDs allows them to be better distributed throughout the epoxy matrix, which, as a result of the formation of chemical interaction at the matrix–nanofiller phase interface, can significantly increase the strength of the obtained composite. The addition of NDs treated with aminoacetic acid ensures an increase in the deformation-strength properties of epoxy composites by 19–23% relative to an epoxy composite containing the pristine NDs. Moreover, the presence of functionalized NDs significantly influences the structure and thermal stability of the epoxy nanocomposite.

## 1. Introduction

Currently, a wide range of polymer composite materials (PCMs) are used to manufacture functional products for various purposes. Among them, epoxy-based composites have become widespread due to their high strength, availability, corrosion resistance, and ease of production. Epoxy-based composites are used in industrial areas such as aerospace, shipbuilding, automotive industry, sporting goods, electronics, etc. However, the requirements for materials are growing, forcing us to look for new ways to improve their properties every year [1,2,3,4,5].

In the last decade, various nano-sized particles used for modifying PCMs became widespread. Such particles include CNTs and MWCNTs [6,7], graphene and graphene oxide [8,9], nanodiamonds [10,11], potassium polytitanates [12,13,14], MXene [15,16], BN particles [17,18], etc. The success of using various nano-sized particles to increase strength and thermal conductivity, change dielectric properties, use as sorbents [19,20,21], as well as to create fireproof materials is confirmed by a large number of studies. The work [8] shows a comparison of the influence of graphene-nanoplateles (Gr) and CNTs on the strength and thermal stability of the resulting epoxy composites. Modification with nano-sized particles increases the tensile strength and impact strength by tens of percent by adding 0.2 to 0.5 wt% Gr and CNTs, depending on the selected characteristics and the type of filler. The authors also note an increase in the density of polymer crosslinking, a decrease in free volume, and restrictions in segmental mobility in combination with better interfacial interaction as a result of the addition of nano-sized particles, which is confirmed by a change in the glass transition temperature by more than 20 °C and a significant change in storage modulus. The effects presented above are achieved by adding a relatively small amount of nanoparticles from 0.01 to 1 wt%. However, the addition of up to tens of percent of nano-sized particles into the matrix can significantly increase thermal conductivity, electrical conductivity, elastic moduli, etc. Neitzel et al. [11] studied the prospects of adding ND in amounts of up to 25 vol.% into the epoxy matrix. They found that the use of 25 vol.% ND increased the elastic modulus and hardness by 470 and 300%, respectively, compared to the original matrix. A significant reduction in the coefficient of friction by 40%, combined with increased thermal conductivity, was noted. The increase in thermal conductivity is explained by the establishment of contact between ND particles with the formation of a developed network held by the matrix inside the polymer.

Among the presented nanoparticles, NDs attract special attention as a modifying matrix component since they have high strength, thermal conductivity, good optical properties, and biocompatibility, combined with availability for production in large volumes at reasonable cost [22]. One of the advantages of NDs is their developed surface, which theoretically allows for increased interaction at the ND–matrix interface, but in practice, it leads to the formation of agglomerates that cannot be dispersed in the volume of the binder by standard methods such as stirring and ultrasonic treatment. As a result, in practice, to reduce the number of agglomerates and the average particle size, surface treatment methods such as energy effects (plasma and UV treatments [23]), physical modification (ball milling [24]), and surface functionalization with various substances are used. The latter method is most widespread, since it allows both reducing the average particle size and establishing the chemical interaction at the phase interface [25,26]. Kim et al. [27] used tetraethylenepentamine to functionalize NDs, which made it possible to achieve an increase in thermal conductivity and fracture toughness by 34 and 121%, respectively, by achieving better dispersion of NDs in the matrix volume, preventing aggregation of nanostructures and building a thermally conductive three-dimensional structure.

In the study [28], a solution of sulfuric and nitric acids was used to functionalize the ND surface, which made it possible to reduce the average size of agglomerates from 300 to 100 nm and of individual particles from 6 nm to less than 4 nm. The authors emphasize that during the functionalization process, sp^2^ carbon is removed from the ND surface and carboxyl and hydroxyl groups are formed that can interact with the epoxy matrix, due to which the greatest increase in tensile strength by 1.5 times at 1 wt% is achieved. Wang et al. [29] assessed the impact on the fire safety and strength of an epoxy composition with ammonium polyphosphate (APP) of the addition of stepwise functionalized ND using P, Cu and (2,2,6,6-tetramethylpiperidin-1-yl)oxyl (TEMPO). The epoxy polymer with 8% APP achieved LOI values of 24.3%, which failed to pass UL-94 V1. The addition of modified NDs made it possible to better remove heat from the surface of the composite due to their high heat transfer and also led to a decrease in the amount of released volatile substances due to the well-known barrier effect of nanomaterials, as well as the formation of a smooth coke layer that prevents the release of flammable substances. The ND modification resulted in an increase in LOI to 28.1%, which corresponds to a fire safety rating of UL-94-V0.

This work explores the influence of two types of nanoparticles of NDs, pristine and aminofunctionalized (using aminoacetic acid), on the kinetics of the curing process, as well as the resulting structure and properties of epoxy nanocomposites. Functionalizing the nanoparticles with aminoacetic acid improved their chemical compatibility with the epoxy composition, making it easier to disperse the nanoparticles. The functionalization of the nanoparticles had a significant impact on the structure, curing kinetics, physicochemical, and mechanical properties of the epoxy nanocomposites. This research is significant in the field of modifying and optimizing the properties of highly efficient and strengthened ND/epoxy nanocomposites.

## 2. Materials and Methods

### 2.1. Materials

Nanodiamonds used as a nanostructuring filler were obtained from the Scientific and Production Closed Joint Stock Company “SINTA”, Minsk, Belarus. Bisphenol-A-based epoxy resin ED-20 and polyethylene polyamine hardener were purchased from CHIMEX Limited, St. Petersburg, Russia. Additionally, 1-chloro-2-propyl phosphate (TCPP) with purity of 95–99%, obtained from VitaReaktiv LLC, Dzerzhinsk, Russia, was used. The presence of phosphorus and chlorine in the TCPP molecule makes it an effective fire retardant. When a composite material containing TCPP undergoes thermal destruction, there is an increase in the production of carbonized structures. These structures act as a barrier, preventing the release of volatile pyrolysis products into the gas phase. As a result, the flammability of the epoxy composite is reduced. This property makes TCPP a valuable ingredient in fire-retardant materials. The information is based on reference [30]. Aminoacetic acid (VitaReaktiv LLC, Dzerzhinsk, Russia) was used to functionalize the ND surface.

### 2.2. Functionalization of the ND Surface

Treatment with aminoacetic acid was carried out with concentrations of 2.5%, 5%, 7.5%, and a ratio of 0.25 g of ND per 50 mL of solution. This technique consisted of the ultrasonic treatment of nanodiamonds in the solution of a selected concentration for 15 min, followed by refluxing at 80 °C for 12 h with stirring at a speed of 100 rpm. Then, the resulting suspension was centrifuged and washed with twice distilled water. The drying process of NDs took place at a temperature of 80 °C for 5 h [31,32].

### 2.3. Characterization of ND

Morphology of ND particles was investigated using a Tescan Vega 3 SBH scanning electron microscope from Brno, Czech Republic. The size distribution of ND particles was determined through laser diffraction using a Zetasizer Nano S instrument from Malvern, Worcestershire, UK. FT-IR spectroscopy of ND samples was performed using a Shimadzu IRTracer-100 machine from Tokyo, Japan. X-ray phase analysis was conducted using an ARL X’tra (Tokyo, Japan) diffractometer with CuKα radiation in the 2θ angle range of 5–60°. Diffraction patterns were interpreted using the International Center for Diffraction Data (ICDD) Powder Diffraction File-2 (PDF-2) database and Crystallographic Search-Match Program, version 3.1.0.2.b. The specific surface area of ND particles was determined through low-temperature nitrogen adsorption using a Quantachrome Nova 2200 (Quantachrome Instruments, Boynton Beach, FL, USA) surface area and porosity analyzer.

### 2.4. Preparation of Epoxy Nanocomposites

The selected number of nanodiamonds was distributed by ultrasonic dispersion at a frequency of 22 ± 2 kHz and a power of 400 W for 60 min, in the composition based on 100 parts by mass of epoxy resin and 40 parts by mass of TCPP (the specified ratio was chosen based on the data presented in [30]). Then, 15 parts by mass of PEPA were added into the resulting composition for curing. All samples were cured at a temperature of 25 ± 2 °C for 24 ± 1 h, followed by stepwise heat treatment at 90 ± 5 °C and 120 ± 5 °C for 2 h.

### 2.5. Testing of the Nanocomposites

Flexural and tensile strength values were obtained in accordance with [33,34] standards, respectively, using a “WDW-5E” testing machine manufactured by Time Group Inc., Beijing, China. The sample testing process took place at a crosshead speed of 5 and 50 mm/min for tensile and bending strength, respectively. The impact strength parameter was determined using an LCT-50D device manufactured by Beijing United Test Co., Ltd., Beijing, China, in accordance with the standard [35]. Vicat heat resistance was determined according to [36], method B50—load 50 N; temperature increase rate 50 °C/h. The processes of curing kinetics were studied by the differential scanning calorimetry (DSC) method using a “DTAS-1300” device (Samara, Russia) under the following conditions: sample weight—20 mg, heating interval—up to 400 °C, heating rate—16 degrees per minute. In addition to the DSC method, a thermometric method, described in detail in [37], was used to study the structure formation processes. Thermogravimetric analysis was performed using the Q-1500D derivatograph system manufactured by MOM in Budapest, Hungary. The samples weighed 100 mg and were heated up to 800 °C at a rate of 10 °C per minute. The analysis was conducted in air, and the relative error did not exceed 1%. This analysis studied the change in mass, rate of mass change, and thermal effects during sample heating.

## 3. Results

**Figure 1:**
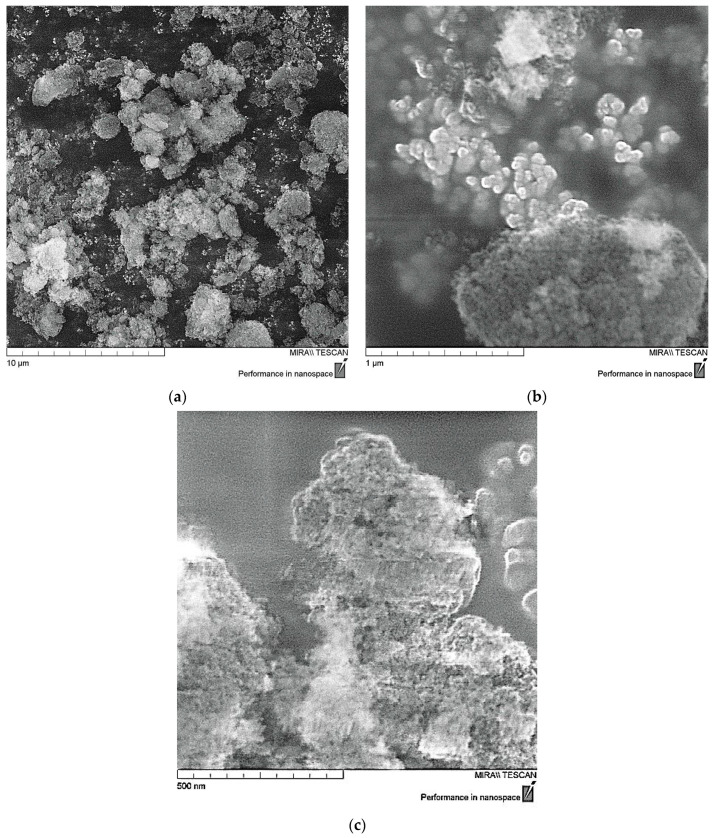
SEM data of ND particles: (**a**)—×10,000; (**b**)—×100,000; (**c**)—×200,000.

The structure of ND nanoparticles was studied by scanning electron microscopy (SEM). Primary nanodiamond particles have a complex structure, close to spherical, as shown in Figure 1. They are a giant molecule in which the carbon part is represented by a diamond core surrounded by a damaged carbon shell which is associated with a layer of surface functional groups localized on the uncompensated valences of surface carbon atoms. The presence of a large number of polar groups in the surface layer of nanodiamonds determines their complex of colloidal chemical properties and also leads to significant agglomeration of particles with the formation of aggregates of 500–1000 nm in size, and it is evident that individual particles have nanosize of 10–50 nm [24,38,39].

**Figure 2:**
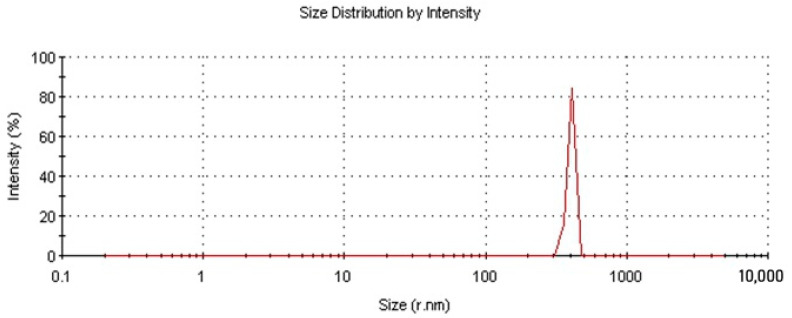
Fractional composition of ND particles.

To determine the average particle size of NDs, the method of laser diffraction in the aqueous solution was used. Figure 2 shows that the size of the pristine nanodiamonds ranged from 300 to 500 nm, with more than 70% of the particles being ~400 nm in size. Using the method of low-temperature nitrogen adsorption, the active surface area of ND was measured to be 292 m^2^/g.

Summarizing the analysis of the pristine NDs, it is necessary to emphasize that the size of individual particles ranges from 10 to 50 nm; however, due to their large active surface area and electrostatic interaction, agglomerates of several hundred nm in size are formed, thereby reducing the potential positive effect of adding NDs into the matrix.

It is well known that NDs have a significant effect on the deformation-strength properties of the resulting nanocomposites. This is usually explained by the formation of a strong transition layer at the interface due to both chemical and physical interaction, which is seen in the so-called nanostructuring effect. However, since NDs are prone to agglomeration because of their highly developed surface, their number in the resulting composite seems to be extremely important.

Table 1 presents data on the deformation-strength characteristics of epoxy nanocomposites, when adding from 0.01 to 0.5 parts by mass of NDs. The analysis of the obtained data shows that the strength of the nanocomposite increases when up to 0.1 parts by mass of NDs are added, after which there is a decrease in the main strength indicators, with an increase in elastic moduli. Modifying the epoxy matrix with 0.1 parts by mass of NDs makes it possible to increase tensile and bending strength by 53 and 30% and the corresponding elastic moduli by 36 and 77%.

The decrease in strength with a further increase in the number of NDs is probably explained by the formation of larger agglomerates, for the separation of which standard processing methods for obtaining nanocomposites are not enough. This assumption is partially confirmed by the high active surface area of the used NDs (292 m^2^/g), as well as by a number of other studies [40,41,42].

Reducing the tendency of nanoparticles to form agglomerates is an extremely important aim of modern polymer science. Currently, the scientific community is inclined to believe that one of the most effective methods for reducing the number of agglomerates in the thermoset matrix is the functionalization of the surface of nano-sized particles with substances capable of chemical interaction with the matrix. This approach ensures their better distribution in the matrix, in combination with strong chemical interaction at the interface, which allows for an increase in the strength of the nanocomposite [43,44]. These substances include aminoacetic acid, which can interact with epoxy units and functional groups of NDs [45,46].

**Figure 3:**
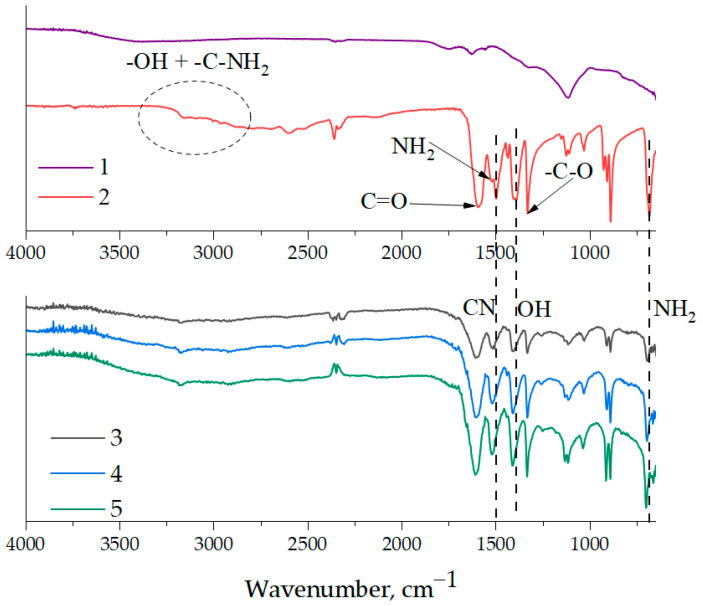
FT-IR spectroscopy of pristine NDs, aminoacetic acid, and ND samples treated with aminoacetic acid: 1—pristine NDs; 2—aminoacetic acid; 3—ND samples treated with 2.5% aminoacetic acid solution; 4—ND samples treated with 5.0% aminoacetic acid solution; 5—ND samples treated with 7.5% aminoacetic acid solution.

Previously published work [31] demonstrated the existence of a chemical interaction between the functional groups of epoxy resin and aminoacetic acid. Figure 3 shows the IR spectra of aminoacetic acid, NDs, and surface-functionalized NDs with different concentrations of aminoacetic acid. The main spectral peaks on the ND surface include the 3430 and 1631 cm^−1^ regions, which are characteristic of stretching and bending vibrations of -OH groups, as well as the 1725 cm^−1^ peak, which belongs to the C=O group. Additionally, 1382 and 1122 cm^−1^ peaks are identified, belonging to the C-H and C-O-C groups, respectively. The presence of functional groups on the ND surface allows us to speak about a potential interaction with aminoacetic acid. As shown in Figure 3, the treatment of NDs with a 5% solution of aminoacetic acid results in the formation of clearly defined peaks associated with it on the surface of nanoparticles, which suggests the success of this process. Separately, it is necessary to highlight NH_2_ groups in the region of 680 and 1500 cm^−1^, capable of interacting with the epoxy units of the polymer matrix.

**Figure 4:**
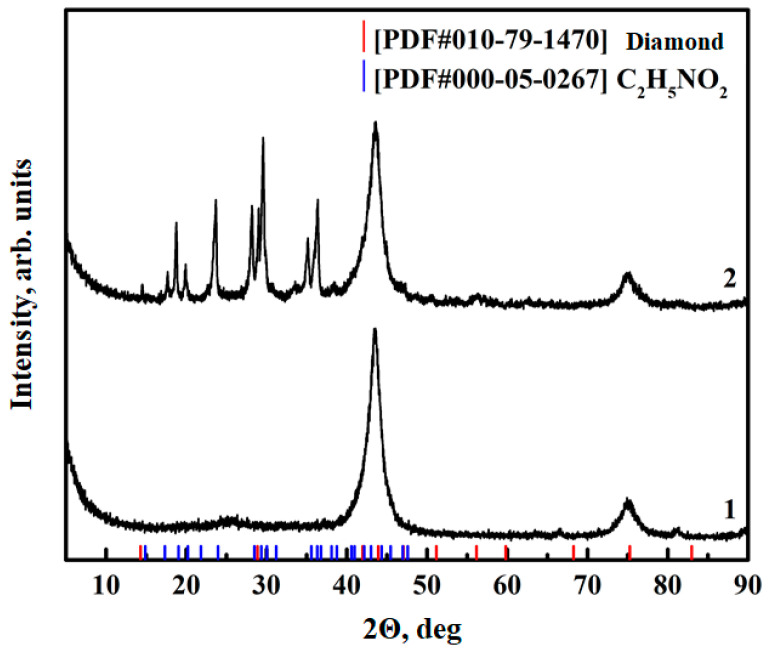
XRF data for ND: 1—pristine ND; 2—after aminoacetic acid treatment.

The presence of functional groups on the ND surface was further confirmed using X-ray diffraction analysis. The XRD diffraction patterns of the pristine NDs, Figure 4, show 43° and 76° (2θ) peaks standard for this material, which are attributable to the diffractions of 111 and 220 planes of a diamond crystal. As a result of the functionalization, the formation of new peaks related to aminoacetic acid was observed while maintaining the structure of NDs, Figure 4, curve 2, which confirms the success of the functionalization.

**Figure 5:**
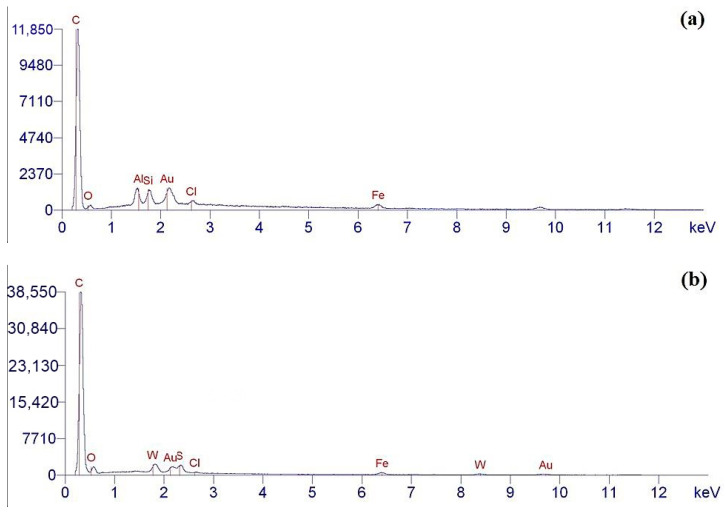
EDX data for ND: (**a**) pristine ND; (**b**) after aminoacetic acid treatment.

The elemental composition of the surface of the pristine and aminoacetic acid-functionalized ND particles was determined by the EDX method. Analysis of the data obtained shows that the chemical composition of the pristine NDs fully corresponds to the composition claimed by the manufacturer, Figure 5a. Using the EDX analysis of the pristine NDs, carbon was detected, which corresponds to the chemical composition of the nanodiamond; additionally, a small amount of oxygen was found on the surface, which corresponds to the presence of hydroxyl groups on the surface of nanoparticles, Figure 5a.

**Figure 6:**
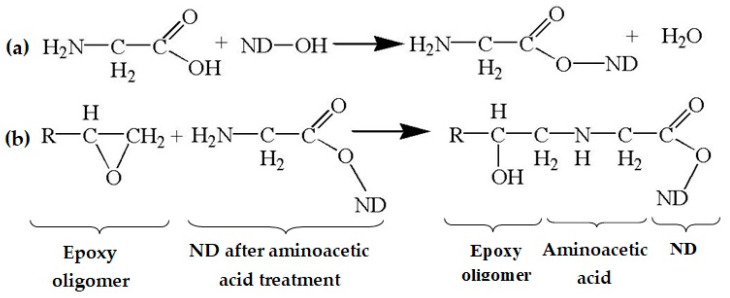
Possible chemistry of the interaction of epoxy oligomer, aminoacetic acid, and ND: (**a**) interaction of aminoacetic acid with ND; (**b**) interaction of ND treated with aminoacetic acid with functional groups of epoxy oligomer.

The elemental composition of NDs functionalized with aminoacetic acid is presented in Figure 5b. Analysis of the obtained EDX data shows the presence of elements such as carbon (C) and oxygen (O); however, the intensity of the peaks is significantly higher than for the pristine NDs, which is ensured by the grafting of aminoacetic acid molecules onto the surface of the ND particles [47,48]. These changes in the spectra of the modified ND particles indicate that the modification was successful, with the fixation of new functional groups capable of chemical interaction with the polymer matrix. Taking into account the data obtained from Fourier infrared spectroscopy, EDX, and X-ray phase analysis, it can be assumed that the reactions presented in Figure 6 occur in the system.

**Figure 7:**
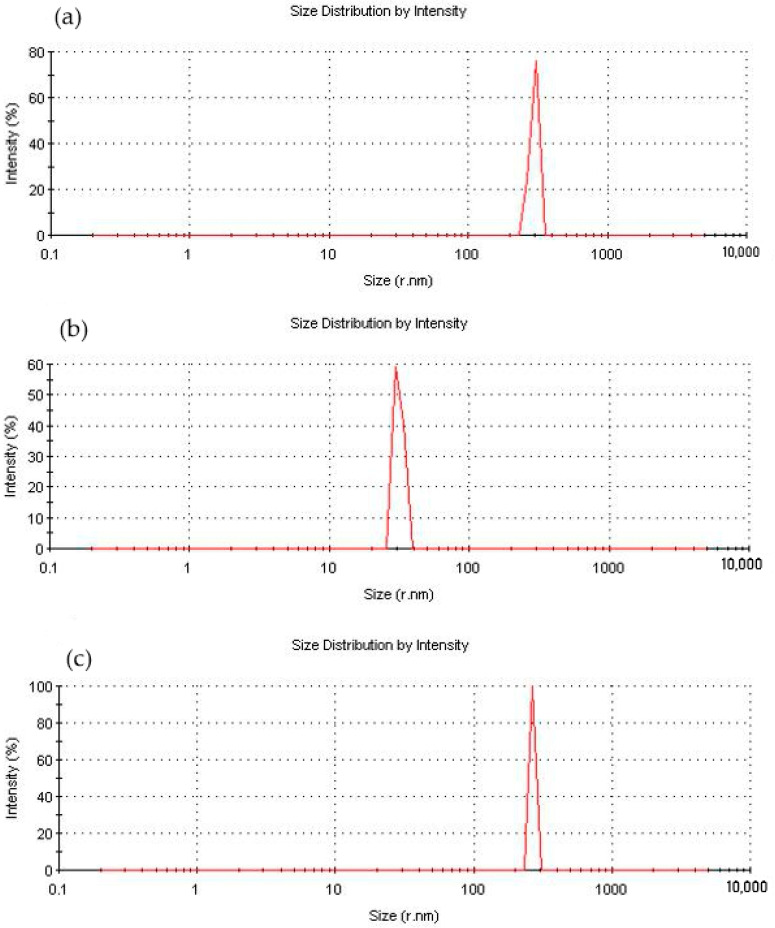
Fractional composition of ND particles modified with aminoacetic acid with different concentrations: (**a**) 2.5%; (**b**) 5%; (**c**) 7.5%.

**Figure 8:**
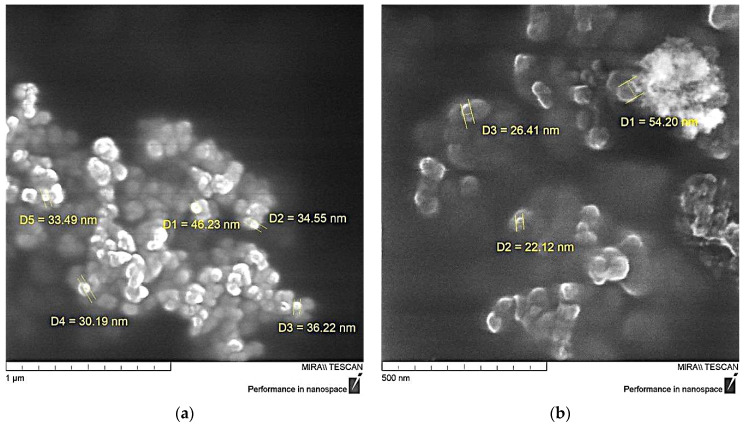
SEM data of ND particles treated with aminoacetic acid: (**a**)—×100,000; (**b**)—×200,000.

Measurement of the particle size of the functionalized NDs showed a significant decrease in their average size, Figure 7, which is likely due to the exfoliation of particles inside the aggregates under the influence of aminoacetic acid and preventing their further self-assembly. The optimal result was achieved when treated with a 5% solution of aminoacetic acid, as a result of which the particle size decreased to 25–40 nm, Figure 7b, which is also confirmed by SEM data, Figure 8. A further increase in the concentration of the aminoacetic acid solution is likely to lead to an increase in the surface interaction, thus resulting in an increase in the average particle size.

**Figure 9:**
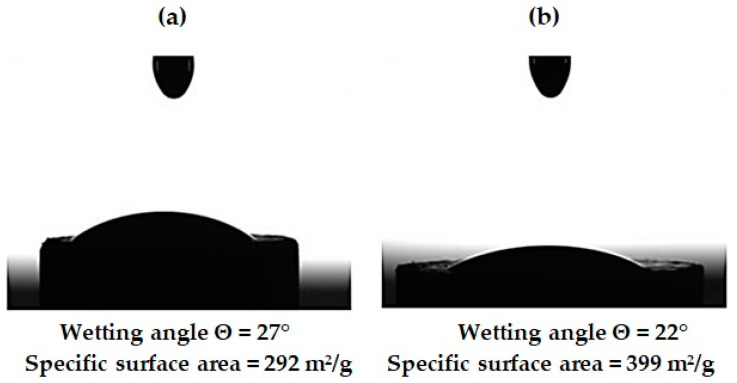
Wetting angle and specific surface area of ND particles: (**a**) pristine ND; (**b**) after aminoacetic acid treatment.

Wetting contact angle and specific surface area of pristine and functionalized NDs were measured as one of the key properties indicative of silane agent grafting. Figure 9 shows the contact angles of nanodiamonds wetting with the epoxy composition used in the work. It was noted that as a result of the functionalization, there was a significant decrease in the contact angle from 27° to 22°, which may be due to an increase in the surface energy of nanoparticles from 292 to 399 m^2^/g as well as the presence of functional groups NH_2_ and COOH on the surface of nanoparticles, capable of interacting with the epoxy composition.

**Figure 10:**
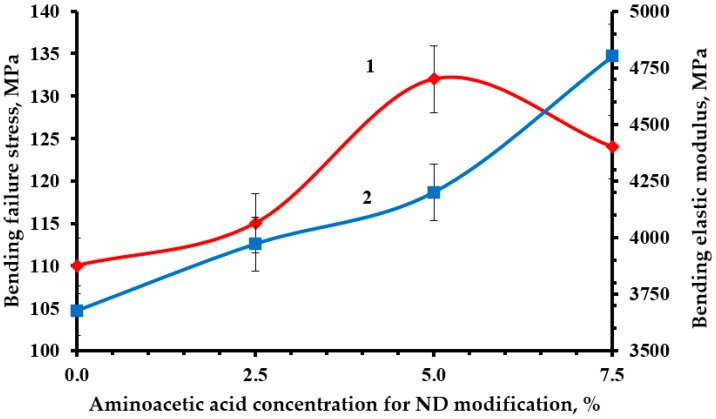
Dependence of bending stress (1) and modulus of elasticity in bending (2) of an epoxy composite containing 0.1 parts by mass of ND on the concentration of aminoacetic acid used to modify NDs.

**Figure 11:**
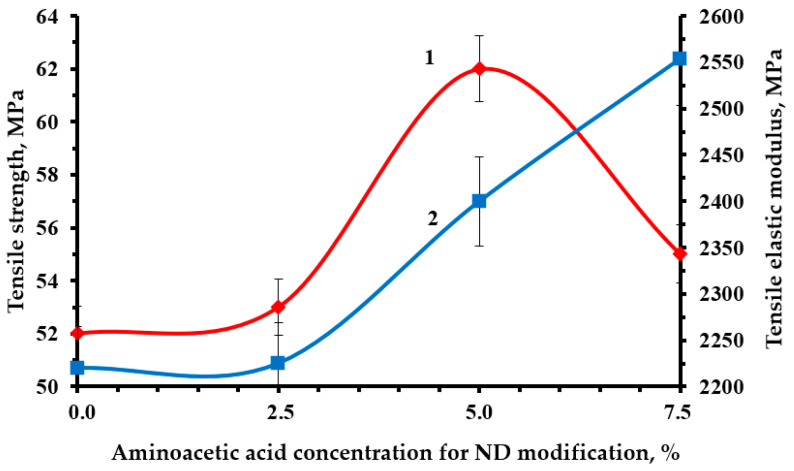
Dependence of the strength (1) and tensile modulus (2) of an epoxy composite containing 0.1 parts by mass of ND on the concentration of aminoacetic acid used to modify NDs.

**Figure 12:**
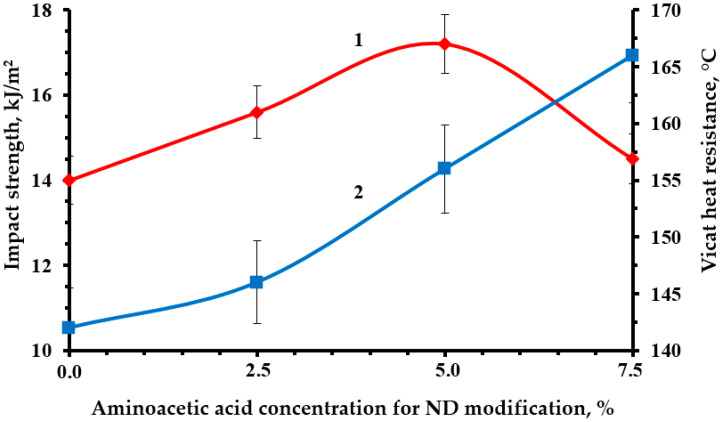
Dependence of impact strength (1) and Vicat heat resistance (2) of an epoxy composite containing 0.1 parts by mass of ND on the concentration of aminoacetic acid used to modify ND.

Optimal strength characteristics were obtained by modifying the NDs with a 5% solution of aminoacetic acid, Figure 10, Figure 11 and Figure 12. The functionalization had a significant impact on the strength indicators, increasing them from 19 to 23% relative to the original nanocomposite. The confirmed presence of functional groups on the ND surface suggests that the strengthening is explained by the formation of a strong interfacial layer capable of fully realizing the nanostructuring effect of NDs. Moreover, a decrease in the average particle size most likely made it possible to better distribute NDs throughout the nanocomposite volume, which led to a decrease in the number of agglomerates and, as a result, a decrease in the number of overstress points that reduce strength.

**Figure 13:**
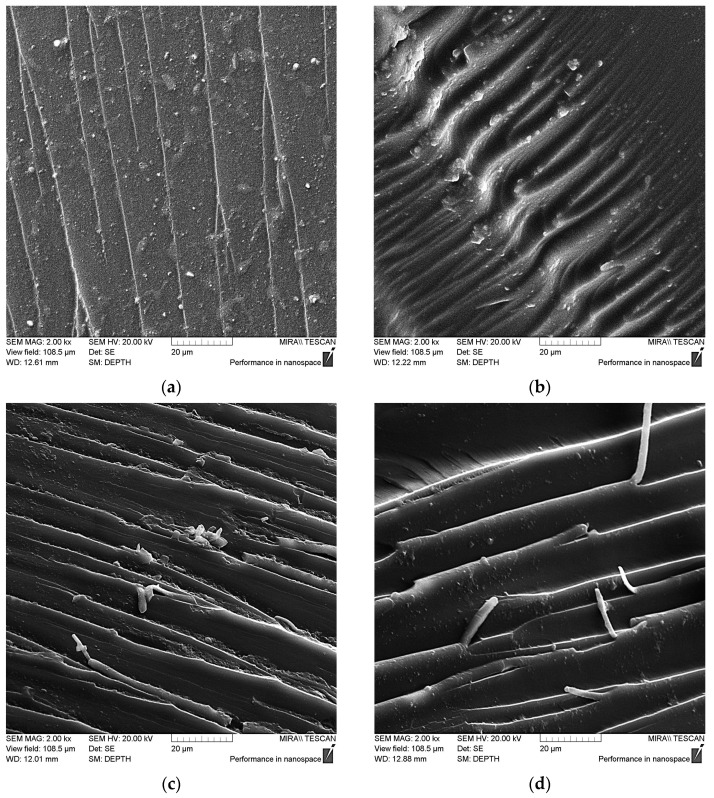
SEM data for epoxy composite samples, parts by mass: (**a**) 100 ED-20 + 40 TCPP + 15 PEPA; (**b**) 100 ED-20 + 40 TCPP + 0.1 ND + 15 PEPA; (**c**,**d**) 100 ED-20 + 40 TCPP + 0.1 ND_(aminoacetic acid)_ + 15 PEPA.

As can be seen in Figure 13a, the pristine epoxy matrix has a relatively smooth chip surface, and the propagation of microcracks is parallel to each other, which together indicates the low energy required to destroy the matrix [49,50]. After adding NDs into the matrix, the nature of the chip changed to a more rigid one, Figure 13b, there was an increase in the number and depth of defects, which indicates the need to spend more energy on the appearance and development of microcracks that reduce the strength of the composite.

The resulting interfacial interaction between the matrix and functionalized NDs, as well as the high surface energy of nanoparticles, leads to a significant change in the nature of the destruction of the composite. This can be seen in Figure 13c,d, where the nature of the destruction changes to numerous tortuous defects with elongated structures at their boundaries. This indicates that nanoparticles taking on the load contribute to the shear of epoxy layers under the influence of mechanical force [51,52]. Plastic deformations noted on the ridges of chips resulting from strong stretching of the nanocomposite confirm the hypothesis that NDs treated with aminoacetic acid can act as a solid-state hardener. This may be on account of the functionalization of the ND surface, which results in the formation of a dense crosslinking network around the nanoparticles of NDs with epoxy groups of oligomer. The combination of these factors allows us to say that an increase in the strength of a given system occurs due to an increase in the energy required for the formation and propagation of microcracks.

The addition of NDs can result in various reactions since a large number of functional groups are present on their surface. Therefore, it is crucially necessary to assess the influence of NDs, as well as their functionalization, on the kinetics of polymer curing processes [53,54,55].

**Figure 14:**
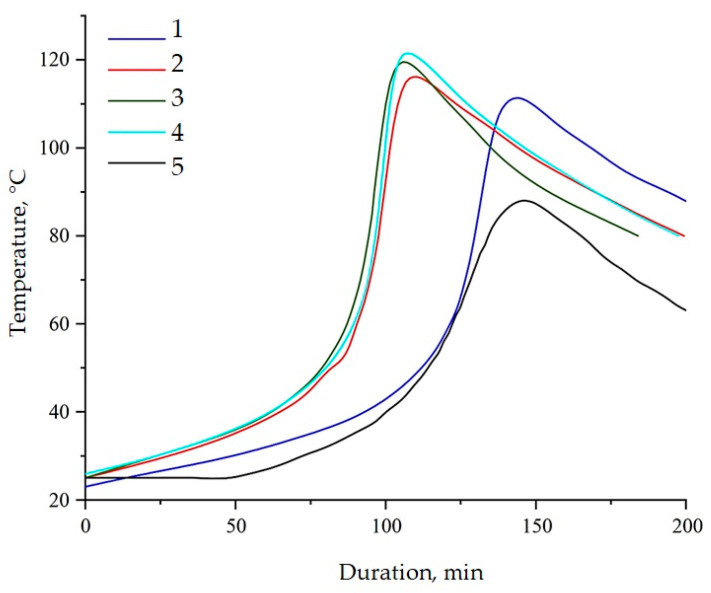
Dependence of sample temperature over time during curing of epoxy compositions containing pristine and modified NDs: (1)—100 ED-20 + 40 TCPP + 0.1 ND + 15 PEPA; (2)—100 ED-20 + 40 TCPP + 0.1 ND_(2.5% aminoacetic acid)_ + 15 PEPA; (3)—100 ED-20 + 40 TCPP + 0.1 ND_(5.0% aminoacetic acid)_ + 15 PEPA; (4)—100 ED-20 + 40 TCPP + 0.1 ND_(7.5% aminoacetic acid)_ + 15 PEPA; (5)—100 ED-20 + 40 TCPP + 15 PEPA.

Epoxy resin is characterized by an exothermic chemical reaction that releases large amounts of energy in the form of heat. Therefore, one of the possible methods for assessing the kinetic processes occurring during the curing process is to construct a graph of self-heating of the composition versus time, carried out under controlled external conditions, Figure 14 and Table 2. Based on this graph, it is possible to establish the gelation time, curing time, and maximum curing temperature. The presented parameters allow us to clearly assess the nature of the influence on the processes of structure formation by modifiers.

The addition of NDs into the epoxy composition led to a decrease in the gelation time from 104 to 95 min and an increase in the maximum curing temperature from 88 to 110 °C. The acceleration of the curing reaction, accompanied by the release of a greater amount of heat, implies the participation of NDs in the polymerization processes. The functionalization of NDs accelerates the curing reaction and increases the amount of heat generated, confirming the fact of chemical interaction between the functional groups of aminoacetic acid and the epoxy matrix [56]. This fact is confirmed by a decrease in the duration of gelation from 95 to 78 min and curing from 142 to 106 min, as well as an increase in the maximum reaction temperature by 12 °C.

**Figure 15:**
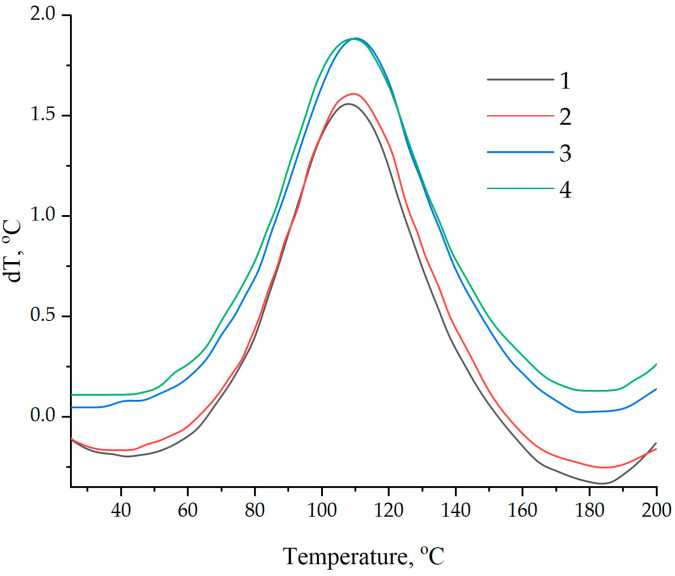
Differential scanning calorimetry of epoxy compositions: (1)—100 ED-20 + 40 TCPP + 0.1 ND + 15 PEPA; (2)—100 ED-20 + 40 TCPP + 0.1 ND_(2.5% aminoacetic acid)_ + 15 PEPA; (3)—100 ED-20 + 40 TCPP + 0.1 ND_(5.0% aminoacetic acid)_ + 15 PEPA; (4)—100 ED-20 + 40 TCPP + 0.1 ND_(7.5% aminoacetic acid)_ + 15 PEPA.

Data on the kinetics of the curing processes were confirmed by DSC, shown in Figure 15 and Table 3. The addition of NDs functionalized with a 5.0% solution of aminoacetic acid into the epoxy matrix increased the reaction enthalpy by more than 40% while reducing the onset temperature of curing from 66 to 41 °C, Table 3.

The analysis of the curing processes of the epoxy composition by the thermometric method and DSC shows that the use of NDs, as well as their further functionalization, intensifies the polymerization reaction process. This fact is probably explained by the participation of functional groups on the ND surface in the interaction with the polymer matrix, as a result of which nanoparticles become additional polymer crosslinking centers. This assumption is confirmed by an increase in thermal effects and the acceleration of the curing reaction as a result of an increase in the concentration of the aminoacetic acid solution treated with NDs.

**Figure 16:**
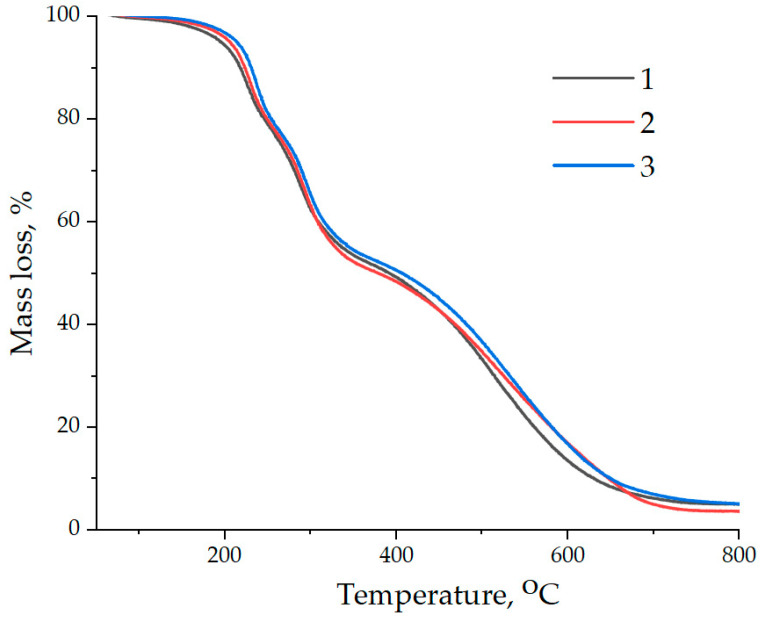
Data of thermogravimetric analysis of samples: (1)—100 ED-20 + 40 TCPP + 15 PEPA; (2)—100 ED-20 + 40 TCPP + 0.1 ND + 15 PEPA; (3)—100 ED-20 + 40 TCPP + 0.1 ND_(5.0% aminoacetic acid)_ + 15 PEPA.

The thermal resistance of epoxy composites was studied by thermographimetry. The obtained data are presented in Figure 16, and are also summarized in Table 4.

Analysis of the obtained data has shown that the addition of NDs functionalized with aminoacetic acid ensures an increase in the initial temperature of destruction of the epoxy composite, which is confirmed by an increase in the T_5%_ index from 195 to 216 °C. Moreover, it has been found that the addition of NDs treated with aminoacetic acid into the epoxy composition increases the thermal stability of epoxy nanocomposites, which is confirmed by an increase in T_30%_, T_50%_, and T_70%_, Table 4.

It has been found that the addition of NDs does not result in a significant increase in the carbon residue at 800 °C, Table 4.

## 4. Conclusions

In summary, NDs were successfully functionalized with aminoacetic acid and then used to prepare nanocomposites. The properties of both pristine and functionalized NDs were studied. Using IR spectroscopy and X-ray phase analysis, the presence of NH_2_ and -COOH was found on the surface of NDs as a result of their functionalization. A decrease in the average particle size from ~400 to 50 nm after the functionalization was shown and the optimal amount of NDs in the epoxy matrix was selected. The functionalization of the ND surface initiated the polymerization processes, reducing the duration of gelation and curing while increasing thermal effects. It has been established that all deformation-strength characteristics increased as a result of the addition of functionalized NDs. SEM data showed changes in the nature of the destruction of the sample from flat, for the pristine epoxy polymer, which has a smooth surface, to uneven with numerous tortuous defects and elongated structures for the nanocomposite with functionalized NDs, which indicates a significantly greater required amount of energy needed for the formation and development of microcracks.

This paper shows that better mechanical properties are achieved when preparing a nanocomposite with 0.1 parts by weight of functionalized NDs. This fact is explained by a decrease in the number of agglomerates and the establishment of a chemical interphase interaction between the matrix and NDs, due to which changes occur in the structure of the nanocomposite, which leads to an increase in its strength. The addition of NDs functionalized with aminoacetic acid ensures an increase in the initial temperature of destruction of the epoxy composite, which is confirmed by an increase in the T_5%_ index from 195 to 216 °C. Moreover, it has been found that the addition of NDs treated with aminoacetic acid into the epoxy composition increases the thermal stability of epoxy nanocomposites, which is confirmed by an increase in T_30%_, T_50%_, and T_70%_.

## Figures and Tables

**Table 1:** Properties of epoxy nanocomposites.

Composition, Parts by Mass	σ_ben_, MPa	E_ben_, MPa	σ_ten_, MPa	E_ten_, MPa	a_im_, kJ/m^2^
100 ED-20 + 40 TCPP + 15 PEPA	85 ± 2.8	2077 ± 62	34 ± 1.7	1634 ± 50	9.0 ± 0.35
100 ED-20 + 40 TCPP + 15 PEPA + 0.01 ND	100 ± 3.0	2734 ± 83	41 ± 2.0	2006 ± 80	9.8 ± 0.38
100 ED-20 + 40 TCPP + 15 PEPA + 0.05 ND	102 ± 3.2	3065 ± 90	46 ± 2.2	2160 ± 86	11.0 ± 0.42
100 ED-20 + 40 TCPP+ 15PEPA + 0.10 ND	110 ± 3.3	3676 ± 105	52 ± 2.4	2220 ± 88	14.0 ± 0.56
100 ED-20 + 40 TCPP + 15 PEPA + 0.50 ND	81 ± 2.5	4668 ± 140	43 ± 2.1	2390 ± 92	10.1 ± 0.40

Note: σ_ben_—bending stress; E_ben_—modulus of elasticity in bending; σ_ten_—tensile strength; E_ten_ is the tensile modulus of elasticity; a_im_—impact strength.

**Table 2:** Values of curing indicators of epoxy compositions.

Composition, Parts by Mass	τ_gel_, min	τ_cur_, min	T_max_, °C
ED-20 + TCPP + PEPA	104	146	88
ED-20 + TCPP + PEPA + ND	95	142	110
ED-20 + TCPP + PEPA + ND_(2.5% aminoacetic acid)_	82	114	115
ED-20 + TCPP + PEPA + ND_(5.0% aminoacetic acid)_	80	108	119
ED-20 + TCPP + PEPA + ND_(7.5% aminoacetic acid)_	78	106	122

Note: τ_gel_—duration of gelation, τ_cur_—duration of curing, T_max_—maximum self-heating temperature of the sample during curing.

**Table 3:** Results of differential scanning calorimetry of epoxy compositions.

Composition, Parts by Mass, Cured by 15 Parts by Mass of PEPA	T_start_–T_end_ T_max_ °C	H, J/g
ED-20 + TCPP + ND	66–151 106	488
ED-20 + TCPP + ND_(2.5% aminoacetic acid)_	64–155 108	585
ED-20 + TCPP + ND_(5.0% aminoacetic acid)_	48–177 110	663
ED-20 + TCPP + ND_(7.5% aminoacetic acid)_	41–175 109	691

Note: T_start_, T_end_—temperature of the start and the end of the curing process, T_max_—the temperature of the maximum heat release during curing, H—thermal effect of reaction.

**Table 4:** The results of the TGA for epoxy nanocomposites.

Composition, Parts by Mass, Cured by 15 Parts by Mass of PEPA	T_5%_, °C	T_30%_, °C	T_50%_, °C	T_70%_, °C	Residues at 800 °C, wt%
ED-20 + TCPP	195	281	392	515	5.1
ED-20 + TCPP + ND	205	284	394	525	3.7
ED-20 + TCPP + ND_(5.0% aminoacetic acid)_	216	291	412	536	5.2

Note: T_5%_, T_30%_, T_50%_, T_70%_—the temperature at a weight loss of 5%, 30%, 50%, 70%, respectively.

## Data Availability

Data are contained within the article.
